# Gastrointestinal disturbance and effect of fecal microbiota transplantation in discharged COVID-19 patients

**DOI:** 10.1186/s13256-020-02583-7

**Published:** 2021-02-08

**Authors:** Fengqiong Liu, Shanliang Ye, Xin Zhu, Xuesong He, Shengzhou Wang, Yinbao Li, Jiang Lin, Jingsu Wang, Yonggan Lin, Xin Ren, Yong Li, Zhaoqun Deng

**Affiliations:** 1 Department of Laboratory Center, The Affiliated People’s Hospital of Jiangsu University, 8 Dianlilu, Zhenjiang, 212000 People’s Republic of China; 2 Ganzhou Municipal Hospital, 49 Dagonglu, Ganzhou, 341000 People’s Republic of China; 3 Department of Epidemiology and Health Statistics, Fujian Provincial Key Laboratory of Environment Factors and Cancer, School of Public Health, Fujian Medical University, Fuzhou, China; 4 School of Pharmacrutical Sciences, Gannan Medical University, Ganzhou, China; 5 GanzhouShanjian Bio-Technology Co., Ltd., Ganzhou, China

**Keywords:** COVID-19, FMT, Microbiome, Infection

## Abstract

**Background:**

To investigate the potential beneficial effect of fecal microbiota transplantation (FMT**)** on gastrointestinal symptoms, gut dysbiosis and immune status in discharged COVID-19 patients.

**Case presentation:**

A total of 11 COVID-19 patients were recruited in April, 2020, about one month on average after they were discharged from the hospital. All subjects received FMT for 4 consecutive days by oral capsule administrations with 10 capsules for each day. In total, 5 out of 11 patients reported to be suffered from gastrointestinal symptoms, which were improved after FMT. After FMT, alterations of B cells were observed, which was characterized as decreased naive B cell (*P* = 0.012) and increased memory B cells (*P* = 0.001) and non-switched B cells (*P* = 0.012).The microbial community richness indicated by operational taxonomic units number, observed species and Chao1 estimator was marginally increased after FMT. Gut microbiome composition of discharged COVID-19 patients differed from that of the general population at both phylum and genera level, which was characterized with a lower proportion of *Firmicutes *(41.0%) and *Actinobacteria *(4.0%), higher proportion of *Bacteroidetes *(42.9%) *and Proteobacteria *(9.2%). FMT can partially restore the gut dysbiosis by increasing the relative abundance of *Actinobacteria *(15.0%) and reducing *Proteobacteria* (2.8%) at the phylum level. At the genera level, *Bifidobacterium* and *Faecalibacterium *had significantly increased after FMT.

**Conclusions:**

After FMT, altered peripheral lymphocyte subset, restored gut microbiota and alleviated gastrointestinal disorders were observe, suggesting that FMT may serve as a potential therapeutic and rehabilitative intervention for the COVID-19.

## Introduction

Fever and cough are the most common clinical manifestations of COVID-19 infection. In addition, the disease can also cause digestive symptoms such as nausea and diarrhea [1, 2], which may be largely underestimated [3]. Apart from these, lymphopenia and hypercytokinemia were also common in COVID-19 patients which suggest that COVID-19 could compromise the immune system [4, 5]. The presence of both lymphopenia and hyper-cytokinemia in COVID-19 patients might indicate the severity of pathogen infection, as previously reported in severe influenza patients during the pandemic of coronavirus (SARS-CoV) in 2003 [6, 7].

Tens of trillions of microbiota are colonized on the mucosal surfaces of the human body such as intestine and respiratory tract. In the past decades, large amount of evidence emerged to support the beneficial effects of commensal bacteria, especially probiotics. In addition to their crucial role in maintaining immune homeostasis of the intestine, studies also reported that commensal bacteria exerts a marked influence on the immune responses at other mucosal surfaces such as the respiratory tract to protect against respiratory influenza virus [8]. Siew C *et al.* observed persistent alterations in the fecal microbiome of SARS-CoV-2 infected patients during the time of hospitalization, which may suggest that targeting gut microbiota is a new therapeutic option or at least is a choice of adjuvant therapy for COVID-19 [9].

Fecal microbiota transplantation (FMT), an effective way to restore gut microbiota [10], was reported to enhance immunity and would be a potential therapy for individuals with pathogen infection [11–14]. Bradley *et al*. reported that antibiotic treatment can reduce intestinal microbiota, thus change the interferon signature driven by commensal in lung epithelia and promote early influenza virus replication in the respiratory tract. The effects can be reversed by FMT [15]. Therefore, it is very likely that FMT can enhance immunity and would be a potential therapy for individuals with virus infection. Given the fact that gastrointestinal symptoms and immunity dysfunction is prevalent in COVID-19 patients, we speculate that FMT can bring beneficial effect on the gut microbiota, gastrointestinal disorders and immunity system after SARS-CoV-2 infection. In this pilot study, we recruited 11 discharged COVID-19 patients in March, 2020 in Jiangxi Province and conducted FMT to investigate the potential benefit effect of FMT on the gut dysbiosis and immune system.

## Methods

### Study participants

**Fig. 1:**
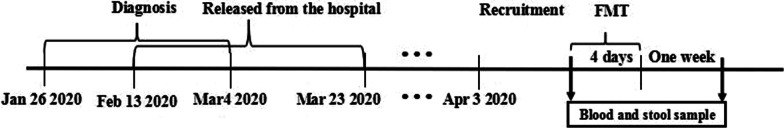
The timeline of patient diagnosis, discharge and recruitment. In total, 11 COVID-19 patients were diagnosed and admitted to the Hospital from January 26 to March 4, 2020. The patients were discharged from hospital from February 13 to March 23, 2020. The recruitment was initiated in April 3, 2020.

This was a prospective, interventional, single-centered pilot study of FMT performed in the Ganzhou city in Jiangxi province of China. The local ethics committee approved the study (Approval number: 2020001) and patients provided written informed consent. In total, 76 COVID-19 cases were confirmed in Ganzhou city since the disease outbreak. In April 2020, we recruited 11 COVID-19 patients who were initially admitted to the Fifth People’s Hospital of Ganzhou City from January 26 to March 4, 2020. The patients were released from hospital from February 13 to March 23, 2020. All the patients were laboratory confirmed positive COVID-19 cases. The timeline of patient diagnosis, discharge and recruitment was summarized (Fig. 1). All the subjects were abstinent from antibiotics or anti-inflammatory drug for two weeks prior to the treatment.

### Data collection

General information included age, sex, occupation, origin, diagnosis date of COVID-19, severity assessment on admission, and the discharged date, which were collected by face to face interview. Intestinal symptoms including constipation, diarrhea, abdominal pain, gastralgia, acid reflux, gastrectasia, as well as fatigue, depression, anxiety, insomnia were obtained by questionnaire.

### Laboratory findings

Laboratory test were evaluated and collected before and after FMT. Laboratory tests included blood routine (leucocytes, neutrophils, lymphocytes, platelets, erythrocyte, haemoglobin), Blood biochemistry (albumin, globulin, A/G, alanine aminotransferase, aspartate aminotransferase, blood urea nitrogen, serum creatinine).

### Lymphocyte subset

Peripheral blood was obtained before and one week after FMT treatment in EDTA tubes for lymphocyte subset detection. All samples were tested within 6 hours after collection. Briefly, 69 indicators were measured by multiple-color flow cytometry with hundreds of human flow monoclonal antibody according to the manufacturer’s instructions. The cells were analyzed on a BDFACS Canto II flow cytometry system (BD Biosciences).The finally lymphocyte subset data was presented as percentage.

### FMT treatment

Protocol of donor screening, FMT capsule preparation, treatment regimen was summarized. Oral capsule administrations were performed in a monitored clinical setting. Participants received 10 capsules each day for 4 consecutive days. The oral capsules prepared for each participant were from a single donor to guarantee the procedure is traceable. Potential side effects such as fever, headache and gastrointestinal symptoms such as diarrhea, nausea, vomiting, distention, abdominal pain were monitored during FMT.

### Gut Microbiome assessments by 16s sequencing

Fecal samples were collected before and one week after FMT. A detailed description of donor stool sampling, 16s sequencing and data processing were summarized. Briefly, sampling packages were distributed to participants and fecal samples were collected and stored in a sealed container which was transported with frozen gel packs to provide a low temperature environment until delivered to the laboratory. Fecal samples were pre-treated and gut microbiome were characterized by 16S sequencing. Original sequencing analysis was performed using QIIME2 and the Silva database was used for taxonomic assignments.

### Statistical analyses

Continuous variable were presented as median and interquartile range. Categorical variable were present as percentage. A paired sample *t*-test was adopted for comparison of variable between pre- and post-FMT for normally distributed data, while Wilcoxon matched-pairs test were performed for data with skewed distribution. The diversity indices evaluating gut microbial community richness (the Chao1 estimator) and alpha diversity (the Shannon and Simpson estimator) were calculated using Mothur.

### Patient and public involvement

Neither patients nor the public were involved in the conception or conduct of the study.

## Results

### Basic information of the 11 COVID-19 patients treated with oral encapsulated FMT

A total of 11 COVID-19 patients who were cured and discharged from the hospital were recruited in the study (Table 1). The median age was 49 years with an interquartile range of 47–57, and 6 patients (54.5%) were male. As for the degree of disease severity on admission, 10 participants were categorized as non-severe. Non-severe was defined as no radiographic evidence of pneumonia or pneumonia was present along with fever and respiratory tract symptoms, but without obvious oxygen saturation change or respiratory failure requiring mechanical ventilation, shock, or organ failure requiring intensive care. One participant was categorized as severe, who suffered from shock and required intensive care.

**Table 1:** Basic information of 11 COVID-19 patients

Patient number	Age, years	Sex	Date of diagnosis	Date of discharge	Severity of Covid-19 on admission
1	57	Male	2020/1/28	2020/2/22	Non-severe
2	51	Female	2020/1/28	2020/2/14	Non-severe
3	45	Male	2020/2/7	2020/2/22	Non-severe
4	49	Male	2020/1/26	2020/2/21	Non-severe
5	49	Female	2020/1/26	2020/2/21	Non-severe
6	23	Female	2020/1/28	2020/2/13	Non-severe
7	48	Male	2020/2/2	2020/2/24	Non-severe
8	47	Male	2020/1/30	2020/2/20	Non-severe
9	58	Male	2020/3/4	2020/3/23	Non-severe
10	68	Female	2020/1/29	2020/2/19	Non-severe
11	53	Female	2020/2/28	2020/3/20	Non-severe

### Gastrointestinal symptoms and lab results of 11 COVID-19 patients before and after FMT

In total, 5 out of 11 discharged patients presented gastrointestinal symptoms to some extent which included constipation, diarrhea, abdominal pain, gastralgia, acid reflux and gastrectasia (Table 2). GI symptoms were present during the admission and persisted beyond discharge. No patient reported GI symptoms before the disease.

**Table 2:** Symptoms of 11 COVID-19 patients before and after fecal microbiota transplantation

Symptoms	Pre-FMT *n*(%)	Symptoms relieved Post-FMT *n*(%)
Constipation	3 (27.3%)	3 (27.3%)
Diarrhea	1 (9.1%)	1 (9.1%)
Abdominal pain	1 (9.1%)	1 (9.1%)
Gastralgia	1 (9.1%)	1 (9.1%)
Acid reflux	2 (18.2%)	1 (9.1%)
Gastrectasia	1 (9.1%)	1 (9.1%)
GI symptoms (in total)	5 (45.5%)	5 (45.5%)
Fatigue	3 (27.3%)	2 (18.2%)
Depression and anxiety	2 (18.2%)	1 (9.1%)
Insomnia	3 (27.3%)	3 (27.3%)
Psychological symptoms (in total)	5 (45.5%)	4 (36.4%)

Values are expressed in number (percentage)

After FMT, 5 subjects reported alleviation in gastrointestinal symptom.

Most of the lab results including blood routine and blood biochemistry were within the normal range in discharged COVID-19 patients (Table 3). 8 out of 11(72.7%) study subjects had mildly decreased Albumin/Globulin ratio, which showed no obvious improvement after FMT.

**Table 3:** Lab results of 11 COVID-19 patients pre- and post-fecal microbiota transplantation

Blood routine	Pre-FMT	Post-FMT
Leucocytes (× 10^9^ per L; normal range 3.5–9.5)	5.6 (4.8, 6.5)	5.9 (4.7, 6.8)
Neutrophils (× 10^9^ per L; normal range 1.8–6.3)	3.2 (2.9, 3.6)	3.3 (2.6, 4.1)
Lymphocytes (× 10^9^ per L; normal range 1.1–3.2)	1.7 (1.6, 2.3)	1.8 (1.6, 2.2)
Monocytes (× 10^9^ per L; normal range 0.1–0.8)	0.4 (0.3, 0.4)	0.4(0.3, 0.5)
Erythrocyte (× 10^12^ per L; normal range 3.5–5.1)	4.8 (4.3, 5.5)	4.8 (4.5, 5.3)
Increased	2 (18.2%)	2 (18.2%)
Haemoglobin (g/L; normal range 120–175)	133 (130, 146)	133 (130, 151)
Increased	1 (9.1%)	1 (9.1%)
Decreased	0 (0.0%)	1 (9.1%)
Platelets (×10^9^ per L; normal range 125–350)	262 (239, 297)	257 (182, 295)
Increased	2 (18.2%)	2 (18.2%)
Blood biochemistry		
ALT (U/L; normal range 9.0–50.0)	18.0 (13.0, 23.0)	15.0 (14.8, 22.0)
AST(U/L; normal range 15.0–40.0)	19.6 (18.0, 20.4)	18.0 (17.0, 21.0)
AST/ALT( normal range 0–3)	1.1 (0.9, 1.5)	1.1 (0.9, 1.4)
Albumin ( 34–54 g/L )	43.0 (40.1, 43.2)	43.0 (42.0, 44.0)
Globulin (20–45 g/L)	30.8 (28.1, 32.3)	30.7 (28.8, 32.4)
A/G ( 1.5-2.5)	1.4 (1.3, 1.5)	1.4 (1.3, 1.5)
Decreased	8 (72.7%)	8 (72.7%)
Blood urea nitrogen (mmol/L; normal range 3.6–9.5)	4.9 (4.0, 5.9)	5.0 (4.5, 5.4)
Increased	1 (9.1%)	1 (9.1%)
Serum creatinine (μmol/L; normal range 57.0–111.0)	77.1 (62.9, 83.0)	79.2 (62.4, 84.0)

Values are expressed in number (percentage) and median (interquartile range)

*FMT* fecal microbiota transplantation, *ALT* alanine aminotransferase, *AST* aspartate aminotransferase, *A/G* albumin/globulin

### Peripheral lymphocyte subset alteration after FMT

In addition to blood routine test, we analyzed lymphocyte subsets composition by flow cytometry. We obtained detailed expression information of 69 different types of lymphocyte and all the lymphocytes were classified into five major subsets, CD4+ T cells (*n* = 17), CD8+ T cells (*n* = 18), γδT cells (*n* = 12),B cells (*n* = 12) and NK cells (*n* = 10). FMT exert significant effect on B lymphocytes which was characterized as decreased naive B cells (*P* = 0.012), increased memory B cells (*P* = 0.001 and non-switched B cells (*P* = 0.012). In addition, the proportion of double positive T cells increased after FMT (*P* = 0.012). γδT cells also showed marginal difference after FMT (Table 4).

**Table 4:** Proportion of lymphocyte subset before and after fecal microbiota transplantation

	Pre-FMT	Post-FMT		*P* value
T cells	64.0 (56.2, 70.9)	62.2 (54.5, 71.6)	% of lymphocyte	0.663
Helper T cells	56.4 (53.8, 66.2)	55.0 (52.0, 62.9)	% of T cells	0.333
Killer T cells	27.1 (22.7, 33.8)	28.2 (24.1, 33.4)	% of T cells	0.062
Double positive T cells	0.8 (0.5, 3.5)	1.1 (0.7, 2.9)	% of T cells	0.012
Th to Tc ratio	2.1 (1.6, 2.5)	2.0 (1.7, 2.5)	–	0.673
γδT cells	1.4 (1.0, 5.3)	4.4 (2.0, 7.6)	% of T cells	0.149
NK cells	13.2 (9.5, 18.7)	13.3 (8.9, 18.6)	% of lymphocyte	0.938
Immature NK cells	5.1 (3.8, 8.4)	7.4 (4.8, 8.3)	% of NK cells	0.936
Mature NK cells	93.3 (91.6, 96.2)	91.9 (91.5, 95.3)	% of NK cell	0.966
Immature/mature NK cells	0.05 (0.04, 0.09)	0.08 (0.05, 0.09)	–	0.905
B cells	10.9 (7.6, 14.0)	8.3 (3.7, 11.5)	% of lymphocyte	0.012
Naïve B cells	62.2 (54.3, 69.1)	40.8 (32.8, 65.2)	% of B cells	0.012
Memory B cells	25.3 (20.2, 30.8)	37.4 (26.3, 48.2)	% of B cells	0.001
Non-switched B cells	10.2 (7.8, 15.2)	21.9 (13.7, 26.0)	% of B cells	0.012
Immature regulatory B cells	0.5 (0.3, 0.8)	1.0 (0.2, 3.0)	% of B cells	0.054

Values are expressed in number (percentage) and median (interquartile range)

*FMT* fecal microbiota transplantation, *T cells* T lymphocyte, *NK cells* natural killer cell, *B cells* B lymphocytes

### Alterations of gut microbiota in discharged COVID-19 patients after FMT

In 22 fecal samples, 970,334 sequencing reads were obtained, and an average of 213 OTUs was identified for each sample. The microbial community richness indicated by OTUs number, observed species and Chao1 estimator was marginally increased after FMT, whereas the alpha diversity estimated by the Shannon and Simpson index showed no significant alteration after FMT (Table 5).

**Table 5:** Community richness and diversity of Gut Microbiota

	Pre-FMT	Post-FMT	*P* value
OTU num	178 (148, 262)	226 (205, 258)	0.101
Observed species	170 (139, 248)	218 (194, 246)	0.100
Chao1 index	225 (182, 305)	293 (257, 327)	0.060
Shannon index	3.78 (3.19, 4.38)	3.41 (3.05, 24.43)	0.800
Simpson index	0.85 (0.80, 0.88)	0.7946 (0.72, 0.89)	0.904

Values are expressed in median (interquartile range)

*FMT* fecal microbiota transplantation, *OTU* operational taxonomic unit

**Fig. 2:**
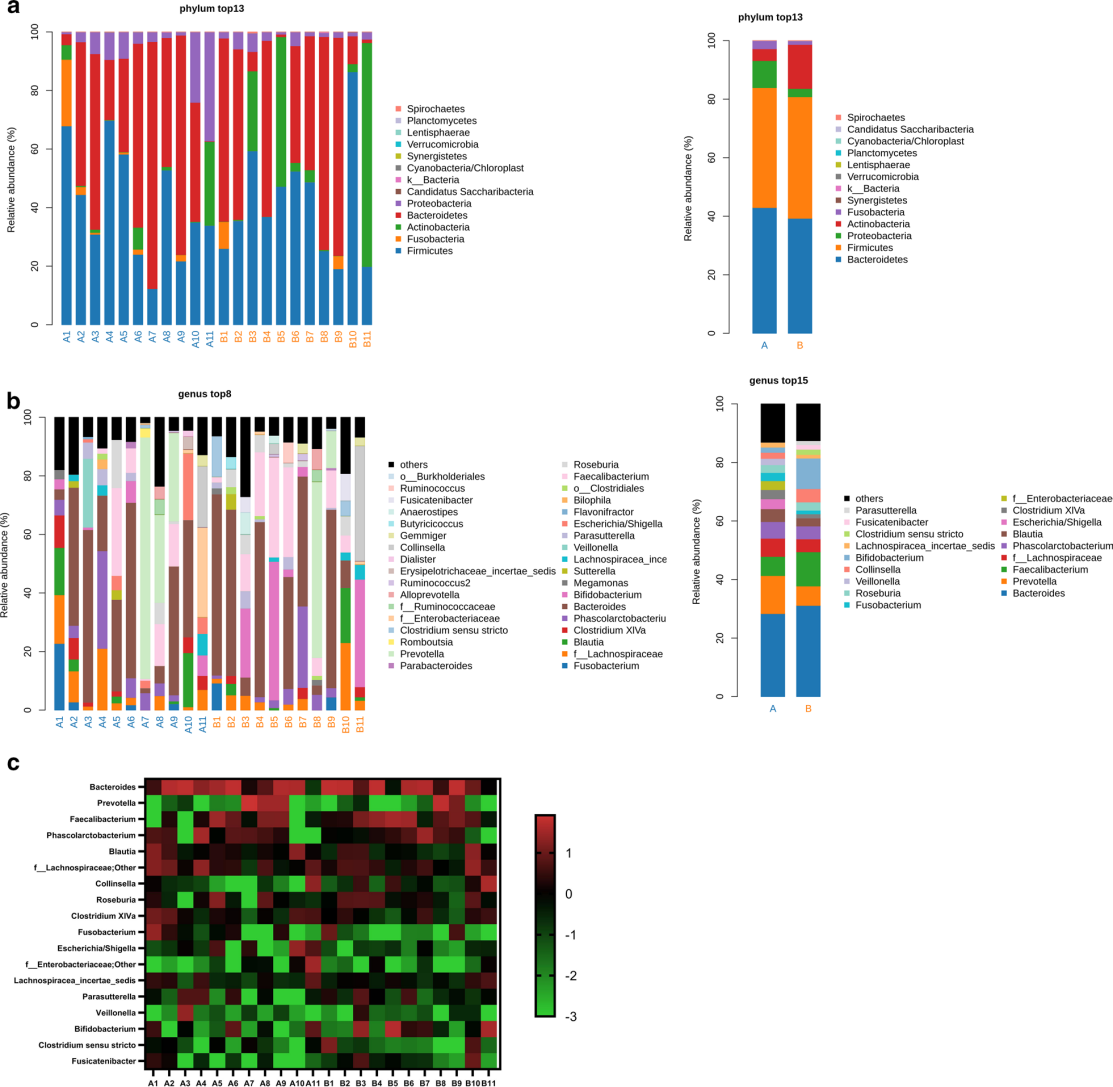
Alterations of gut microbiota in discharged COVID-19 patients after FMT. Alterations of gut microbiota were presented at the phylum and genera level. **a** Changes of relative abundance of individual phylum for both individual patients and at average level were presented. **b** Changes of relative abundance of individual genus for both individual patients and at average level were presented. **c** Heatmap of changes in relative abundance of the top 15 genera. A^#^ represent pre-FMT, B^#^ represent post-FMT.

At the phylum level, the top 5 phylum at baseline include *Firmicutes *(41.0%), *Bacteroidetes *(42.9%), *Proteobacteria* (9.2%),*Actinobacteria*(4.0%),

*Fusobacteria *(2.8%). The top 5 phylum after FMT included *Firmicutes *(41.5%), *Bacteroidetes *(39.3%),*Actinobacteria*(15%), *Proteobacteria *(2.8%),

*Fusobacteria *(1.3%). The relative abundance of *Proteobacteria* decreased, while *Actinobacteria* increased after intervention (*P*<0.001) (Fig. 2).For individual patient, patient No. 1, who had moderate constipation but greatly improved after FMT, was characterized with high proportion of *Firmicutes*(67.8%) and *Fusobacteria *(22.7%)and absence of *Bacteroidetes* (3.7%). FMT significantly increased *Bacteroidetes *(62.6%), decreased *Firmicutes *(26.0%) and *Fusobacteria *(9.2%). Patient No. 7 was a severe COVID-19 survivor who suffered from diarrhea. The patient presented a microbiome profile of extremely high *Bacteroidetes *(84.4%) and low relative abundance of *Firmicutes *(12.3%). After FMT, the proportion of *Bacteroidetes *(45.7%) decreased and *Firmicutes* increased (48.7%). Patient No.11 suffered from severe constipation, gut microbiota profile of whom showed high proportion of *Actinobacteria *(28.6%) and *Proteobacteria *(37.2%), whereas low abundance of *Bacteroidetes *(0.2%). Significant decrease in abundance of *Proteobacteria *(2.5%) was observed after FMT.

At the genera level, the top 5 genera before FMT included *Bacteroides *(28.3%), *Prevotella *(13.0%), *Faecalibacterium *(6.5%), *Lachnospiraceae *(6.2%), *Phascolarctobacterium*(5.7%) at baseline, while after FMT the top 5 genera include *Bacteroides *(31.1%),*Faecalibacterium*(11.7%), *Prevotella *(6.6%), *Bifidobacterium* (10.4%), *Collinsella *(4.5%) (Fig. 2). *Bifidobacterium*, *Faecalibacterium*, *Collinsella* significantly increased after FMT. For individual patient, patient No. 1 was characterized with high proportion of *Fusobacterium *(22.7%), *Lachnospiraceae* (16.6%), *Blautia *(16.1%), *ClostridiumXlVa* (11.1%). After FMT, *Bacteroides *(61.9%) increased, whereas proportions of *Blautia *(0.25%), *ClostridiumXlVa *(1.0%), *Lachnospiraceae* (1.6%), *Fusobacterium *(9.2%) were decreased. Patient No.7 with severe COVID-19 presented high proportion of *Prevotella *(82.5%) which was decreased (0.01%), while *Bacteroides* (44.4%) and *Phascolarctobacterium* (27.8%) increased after FMT. Patient No.11 had high proportion of *Enterobacteriaceae* (30.7%) and *Collinsella* (20.9 %). After FMT, relative abundance of *Enterobacteriaceae* decreased (1.2%), while *Bifidobacterium* significantly increased (36.7%).

### Side effects of FMT

Potential side effects included gastrointestinal symptoms such as diarrhea, abdominal pain were monitored during FMT.

### Informed consent

Written informed consent was obtained from the patient for publication of this case report and any accompanying images. A copy of the written consent is available for review by the Editor-in-Chief of this journal.

## Discussion

We are for the first time followed up COVID-19 patients after they were cured and discharged from hospital and observed that even in the discharged COVID-19 patients, problems such as gastrointestinal and psychological disorder, compromised immunity, gut dysbiosis are prevalent. Altered peripheral lymphocyte subset, restored gut microbiota and alleviated gastrointestinal disorders were observe after FMT.

Early reports showed that 2–14.7% of SARS-CoV-2 infected patients had symptoms of diarrhea and 1–5% of the case had nausea and vomiting. Recently, Cheung *et al*. published a meta-analysis to report that up to 20% had gastrointestinal symptoms [3, 4, 16–18]. Fecal samples from about 50% of COVID-19 patients were detected as SARS-CoV-2 positive, suggesting that the digestive tract might be another site for virus replication and activity [19, 20]. However, report about the after-effect of COVID-19 patients is rare. We are among the first to focus on the rehabilitation patient and report that even in the discharged COVID-19 patients, problems such as gastrointestinal disorder are not uncommon. However, these problems are largely underestimated and neglected. The latest COVID-19 report from China observed that up to 3 % of discharged patients were tested positive on a retest for SARS-CoV-2 and 35% of them had at least one symptom associated with active COVID-19 [21]. Thus, with the rapidly rising number of recovered patients, more and more attention will be paid to the health conditions of patient after the disease.

It has long been reported that respiratory viral infections can lead to alterations in gut microbiome, and gut microbiome disturbance would predispose patients to secondary bacterial infections [22, 23]. For COVID-19, the angiotensin converting enzyme 2 (ACE2) was reported to be a key receptor which facilitate the coronavirus to enter the host. ACE2 is not only expressed in respiratory tract, but also highly expressed in the gastrointestinal tract [24, 25], which may partly explained the gastrointestinal symptoms presented in COVID-19 patients. To date, the only direct evidence links COVID-19 to gut microbiota was reported by Siew *et al*., who investigated changes of fecal microbiomes of COVID-19 patients during hospitalization. Persistent alterations of fecal microbiome were observed in hospitalized patients. Fecal microbiota alterations were positively associated with fecal virus load of SARS-CoV-2 and the disease severity of COVID-19 [9]. In the current study, instead of focusing on hospitalized patients, we followed up COVID-19 patients and observed persistent changes in the fecal microbiome composition after they were cured and discharged from hospital. At the phylum level, the relative abundance of *Firmicutes*, *Bacteroidetes*, *Actinobacteria*, *Proteobacteria *were 41.0%, 42.9%, 9.2%, 4.0% respectively, which were different from that of the general population. In health population, the dominant phyla are *Firmicutes* and *Bacteroidetes *with a relative abundance of about 60% and 20% respectively [26–28]. As for *Actinobacteria* and *Proteobacteria*, data from Asia population reported that the relative abundance was within the range of 0.12–0.22% and 0.01–0.03% respectively [29].

As Siew *et al*. reported in their study that targeting the intestinal microbiota might reduce disease severity of COVID-19 [9]. Actually, at the beginning of February 2020, the guidance of China’s National Health Commission (5th edition) recommended that probiotics can be used to maintain the intestinal microecological balance and prevent secondary bacterial infection when treating patients with severe COVID-19 infection. We further investigate whether FMT could be an effective strategy to improving the residual effect of COVID-19 by modifying the gut microbiome. We observed gut microbiome alteration and symptom alleviation after FMT, especially in patients with severe gastrointestinal symptoms. At the phylum level, the relative abundance of *Actinobacteria *(15%) and *Proteobacteria *(2.8%) were restored to the average level of the general population reported [29]. At the genera level, *Bifidobacterium* and *Faecalibacterium* significantly increased after FMT, especially in those COVID-19 patients with diarrhea or constipation. *Bifidobacterium* and *Faecalibacterium* are both dominant genera in human gut microbiota and are closely related to gut health [30–32].

Gut microbiota could not only maintain immune homeostasis and immune responses at local mucosal surfaces, but also has distal protective effects and protect against respiratory influenza virus. Several studies have reported the application of FMT to improve immune functionality, thus exert indirect protective effect on virus influenza infection. Bradley *et al*. reported that antibiotic treatment can reduce intestinal microbiota, thus change the interferon signature driven by commensal in lung epithelia and promote early influenza virus replication in the respiratory tract. The effects can be reversed by fecal transplantation [15]. Tiffany *et al*. conducted FMT experiments on rhesus monkeys infected with chronic SIV during antiretroviral therapy. After antibiotic treatment, greatest microbiota shift was observed, while the frequencies of Th17 and Th22 in peripheral blood increased and the activation of CD4 T cells in intestinal tract decreased after FMT [33]. The latest evidence from Yongxi Zhang *et al*. reported persistent alterations of peripheral lymphocyte subset in COVID-19 patients, which confirmed the immunity dysfunction after SARS-CoV-2 infection [34]. In the current study, we also observed that the general distribution of 69 different types of lymphocytes differed between Pre-FMT and Post-FMT especially for B lymphocyte subset, which suggest targeting gut microbiota by FMT have favorable effects on the immunity system after SARS-CoV-2 infection.

### Limitations of this study

One major limitation of this exploratory study is the limited sample size. Although the association between SARS-CoV-2 infection and gastrointestinal symptoms, gut dysbiosis in discharged patients requires validation from large scale studies, this pilot study for the time examined the after effect of SARS-CoV2 infection which include gastrointestinal symptoms, peripheral lymphocyte alteration and gut dysbiosis. Another major limitation is that the study is not randomized designed. Although establishing a causative relationship between FMT and gut microbiota regulation in discharged patients requires a parallel control group, it is the first time to examine the effect of FMT on the residual symptoms of SARS-CoV2 infection, and refer to FMT as a potential therapeutic and rehabilitative intervention for the COVID-19. We also attempted to evaluate the immune status and justify the beneficial effects of FMT from the perspective of immunity improvement. Further large scale studies with a randomized design to delineate the role of FMT and microbiome changes in SARS-CoV-2 infection and post-infection recovery.

## Conclusions

Gastrointestinal and psychological symptoms, gut dysbiosis were observed in COVID-19 patients during post-infection recovery. After FMT, altered peripheral lymphocyte subset, restored gut microbiota and alleviated gastrointestinal disorders were observe, suggesting that FMT may serve as a potential therapeutic and rehabilitative intervention for the COVID-19.
